# Two-dimensional radial-π-stacks in solution†

**DOI:** 10.1039/d4sc00195h

**Published:** 2024-03-11

**Authors:** Feng Su, Yongseok Hong, Guilan Zhang, Kongchuan Wu, Juno Kim, Zhi Chen, Hui-Jun Zhang, Dongho Kim, Jianbin Lin

**Affiliations:** a Department of Chemistry, College of Chemistry and Chemical Engineering, MOE Key Laboratory of Spectrochemical Analysis and Instrumentation, Xiamen University Xiamen 361005 P. R. China; b Department of Chemistry, Yonsei University Seoul 03722 Korea; c College of Chemistry and Environmental Engineering, Shenzhen University Shenzhen 518060 China; d Division of Energy Materials, Pohang University of Science and Technology (POSTECH) Pohang 37673 Korea

## Abstract

Highly organized π-aggregate architectures can strongly affect electronic couplings, leading to important photophysical behaviors. With the escalating interest in two-dimensional (2D) materials attributed to their exceptional electronic and optical characteristics, there is growing anticipation that 2D radial-π-stacks built upon radial π-conjugation nanorings, incorporating intra- and inter-ring electronic couplings within the confines of a 2D plane, will exhibit superior topological attributes and distinct properties. Despite their immense potential, the design and synthesis of 2D π-stacks have proven to be a formidable challenge due to the insufficient π–π interactions necessary for stable stacking. In this study, we present the successful preparation of single-layer 2D radial-π-stacks in a solution. Pillar-shaped radially π-conjugated [4]cyclo-naphthodithiophene diimide ([4]C-NDTIs) molecules were tetragonally arranged *via* in-plane intermolecular π–π interactions. These 2D π-stacks have a unique topology that differs from that of conventional 1D π-stacks and exhibit notable properties, such as acting as a 2D template capable of absorbing C_60_ guest molecules and facilitating the formation of 2D radial-π-stacks comprising [4]C-NDTI-C_60_ complexes, rapid exciton delocalization across the 2D plane, and efficient excitation energy funneling towards a trap.

## Abstract 2

We present the formation of 2D radial-π-stacks, consisting of radial π-conjugation nanorings in solution, characterized by their distinctive structure and remarkable properties.

## Graphical abstract

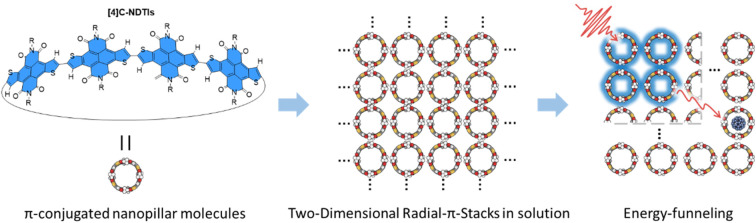

## Introduction

Since the discovery of graphene, two-dimensional (2D) nanomaterials have been a topic of significant research interest due to their unique physical and chemical properties, such as large specific surface areas, excellent optical transparency, and superior electrical and thermal conductivity.^1,2^ Among these materials, those consisting of distinct compositions connected by covalent bonds into a sheet-like morphology have especially piqued interest.^3–5^ However, achieving the desired material properties often relies on non-covalent π stacking interactions, which play a crucial role in material construction^6–9^ and coherent energy transport.^10–12^ For instance, by means of π–π stacking interactions, π-systems are stacked into one-dimensional (1D) columnar π-stacks, which facilitate directional energy transport and pave the way for the development of organic electronic and photonic materials.^13–17^ To this end, there is a pressing need to develop effective strategies for densely packing specific π-systems into precisely ordered 2D π-stacks, which represents a promising direction for the design and synthesis of novel 2D materials.

In nature, with the use of proteinaceous scaffolding, purple bacteria have evolved cyclic BChl-protein aggregates that further assemble into exquisite 2D arrays. Such 2D arrays of circular rings ensure a steady supply of excitation energy by enlarging the absorption cross-section (∼100 fold), rapidly (within picosecond time scale) and efficiently (>95%) funneling the excitation energy to the reaction center (RC).^18–21^ Despite the desire to synthesize 2D π-stacks, this presents a formidable challenge due to the conflicting characteristics required for monomers to propagate the assembly in a 2D plane. With cyclic arrays being a natural choice, rigid and cyclic π-systems can arrange in a lattice packing that forms a 2D well-ordered network. To achieve π-conjugation throughout the loop, it is necessary to directly catenate the aromatic rings into strained π-conjugated nanoring molecules with symmetric rings containing identical sites.^22–25^ This arrangement allows for the formation of circular exciton pathways, as observed in nanoring molecules, such as butadiyne-bridged porphyrin nanorings^26^ and [*n*]cycloparaphenylenes (CPPs)/CPP analogues.^27–33^ However, cyclic π-skeletons with ring tension have minimal contact areas with neighboring molecules in a convex–convex arrangement, which is similar to the interactions between fullerenes (C_60_), thus making it inadequate to maintain 2D stacks.^34^ Although rare instances of reported 2D networks have been observed in cyclophanes,^35,36^ pillar[*n*]arenes,^37,38^ and pagoda[*n*]arenes^39^ in the solid state,^40^ conjugation is sacrificed in these structures by isolating the repeating π-units with “–CH_2_–” bridges to maintain planar interacting π-surfaces. More importantly, it is imperative to study the exciton coupling properties of 2D π-stacks in solution,^41^ which presents additional challenges since the dispersion forces between pigment molecules that drive aggregation can be largely offset by pigment–solvent dispersion forces.

**Scheme 1:**
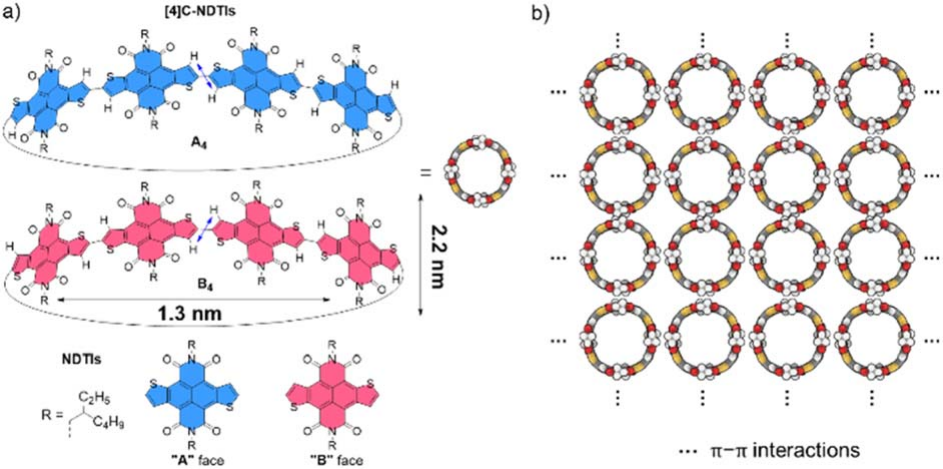
(a) The enantiomers of [4]C-NDTIs: A_4_ and B_4_; the orientation of the NDTI units marked as A and B denoted with blue and red, respectively (diameter and height values of the nanopillar molecule based on the crystal structure). (b) 2D radial-π-stacks based on [4]C-NDTIs.

Recently, we embarked on a program aimed at challenging this notion by designing and synthesizing pillar-shaped radially π-conjugated molecules, [4]cyclo-naphthodithiophene diimides ([4]C-NDTIs) (Scheme 1a).^42^ In comparison to distorted CPP analogues, the thiophene–thiophene connection between adjacent NDTI units imparts strong electronic couplings within the ring structure of [4]C-NDTIs. Furthermore, the intermolecular interactions between the donor (thiophene) and acceptor (naphthalenediimide) moieties of the large, rigid NDTI units promote “exo-wall” convex–convex π–π interactions. In this article, we present the successful development of [4]C-NDTIs based 2D radial-π-stacks with a high degree of order and long-range periodicity in solution (Scheme 1b). The radially aligned 2D structures boast highly organized arrangements and possess the remarkable capacity to encapsulate guest molecules within the hollow cavity of [4]C-NDTIs. The strong exciton coupling in the 2D plane promotes the delocalization of excitons, ultimately leading to efficient charge transfer by channeling the excitation energy to the trap. The remarkable topology and significant characteristics of these 2D π-stacks are reminiscent of the sophisticated light-harvesting (LH) systems and offer promising prospects for the development of functionalized electronic devices based on in-plane 2D materials.

## Results and discussion

### 2D radial-π-stacks

**Fig. 1:**
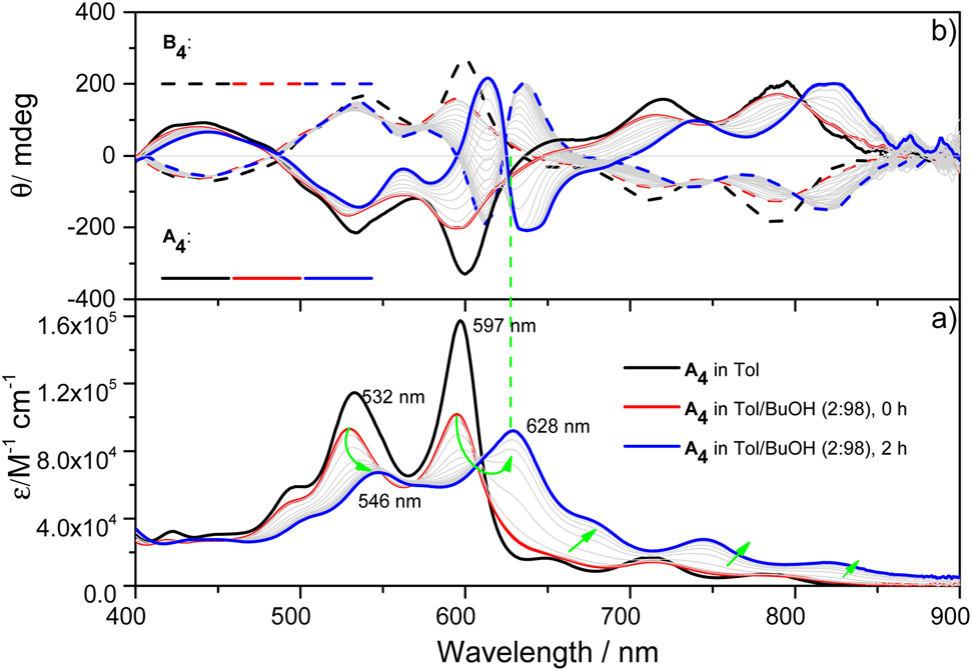
(a) UV-Vis-NIR absorption and (b) CD spectral changes for 2 h when 60 μL of A_4_/B_4_ stock solution at 5 × 10^−4^ M in Tol was added into 2940 μL of *n*-BuOH at 298 K. (Arrows indicate the spectral changes with time).

**Fig. 2:**
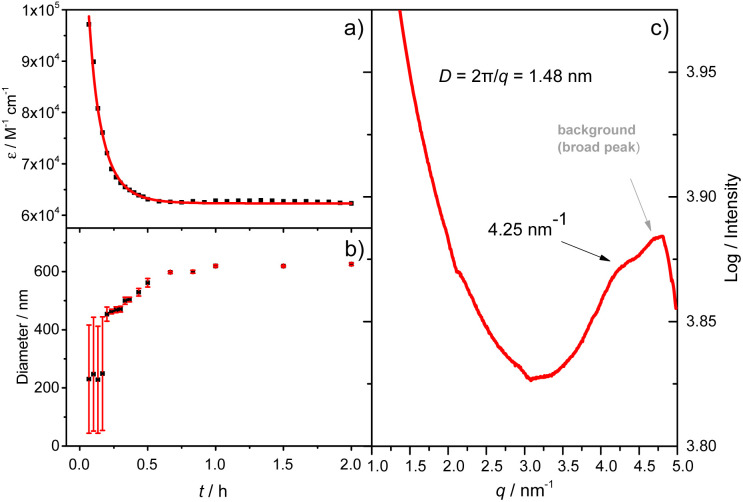
(a) A plot of the absorbance at 597 nm *versus* time and the best fitting to a simple mono-exponential equation; (b) evolution of the aggregate size as a function of time obtained by DLS (the average value over the time range is highlighted with a horizontal bar. Error bars represent the standard deviation from at least 3 measurements); (c) solution-phase small-angle X-ray scattering profile of the A_4_ aggregates (the broader diffusion signal is from the Kapton background). (Tol/*n*-BuOH = 1/49, v/v, 10 μM).

X-ray single-crystal structural analysis of [4]C-NDTIs has indicated the formation of 2D layers through “exo-wall” π–π interactions in the crystalline state.^42^ Normally, π-stacking of such a π-conjugated system is favored in a nonpolar solvent methylcyclohexane (MCH). However, [4]C-NDTIs are molecularly dissolved in MCH based on the results of temperature- and concentration-dependent spectral analyses. To this end, we tried to augment the π–π interactions by utilizing the hydrophobic effect. Among the six isomers of [4]C-NDTIs, one set of enantiomers A_4_ and B_4_ (Scheme 1a) were studied here. Concentrated solution of molecularly dissolved A_4_/B_4_ in toluene (Tol) was injected into an excess of *n*-butanol (*n*-BuOH) to initiate self-assembly. The subsequent formation of 2D-stacks was monitored by UV-Vis-NIR and circular dichroism (CD) spectroscopy. As shown in Fig. 1a, at an optimum volume ratio (Tol/*n*-BuOH = 1/49, v/v), A_4_ molecules assembled with a peculiarity of time dependency. In Tol, A_4_ was molecularly dissolved and displayed two absorption maxima at 597 and 532 nm.^42^ After mixing with *n*-BuOH, the absorption and CD variations were recorded in time intervals of 2 min within the first 30 min and then in time intervals of 5 min within the next 90 min. The absorption band structures became gradually red-shifted and broadened (*i.e.* 597 and 532 nm to 628 and 546 nm, respectively), suggesting strong and complex electronic coupling through the self-assembly process. The isosbestic points at 608 and 570 nm also suggest the gradual transition from monomeric to aggregate states. The measured absorption coefficient values at 597 nm against time could be fitted as a single first-order process (*k* ≈ 9.4 h^−1^, Fig. 2a).^43^ The observed result aligns with the kinetics of homogeneous 2D polymer formation, where growth dominates over nucleation as monomers reach low concentrations. The growth process also exhibits a first-order dependence on monomer concentration.^44^ Although the absorption spectrum of A_4_ aggregates is too complex to have a delicate analysis, the equivalent ratio of *A*_597 nm_/*A*_532 nm_ (monomeric state) and *A*_628 nm_/*A*_546 nm_ (aggregate state) = 1.37 suggests destructive interference between dipole (short-range) and orbital (charge-transfer mediated coupling) interactions,^45–47^ and the formation of A_4_ aggregates *via* novel electronic coupling.

As shown in Fig. 1b, the mirror-image CD spectra between A_4_ and B_4_ exhibit gradual bathochromic shifts over time, which are fully developed within 30 min and remained constant in the next 90 min. The A_4_ and B_4_ aggregates show strong bisignate cotton coupling between the π-skeletons, indicating the formation of π-aggregates. Aggregate size monitoring by dynamic light-scattering (DLS) confirmed a constant particle growth after initiation. Within approximately 30 min, the aggregate size reached a plateau at ∼600 nm in diameter (Fig. 2b). The 2D structure in Tol/*n*-BuOH solution was further revealed by a solution-phase small-angle X-ray scattering (SAXS) experiment.^48^ As shown in Fig. 2c, a scattering peak corresponding to a *d* space of 1.48 nm is observed, which is consistent with the expected pore diameter calculated on the basis of the crystal structure of A_4_ and clearly indicated the presence of the 2D periodic array in solution. Due to the π–π stacking, the fluorescence of A_4_ in Tol/*n*-BuOH solution displays strong quenching (Fig. S1†). These collective findings support that A_4_ and B_4_ form chiral and radial aggregates in the mixed solvent through π-stacking. Considering the *D*_4_ symmetry of A_4_ and B_4_ with nanopillar conformations, it is highly possible that the π stacking guides an ordered elongation of [4]C-NDTIs to form 2D radial-π-stacks as the single layers in the solid state.^42^

**Fig. 3:**
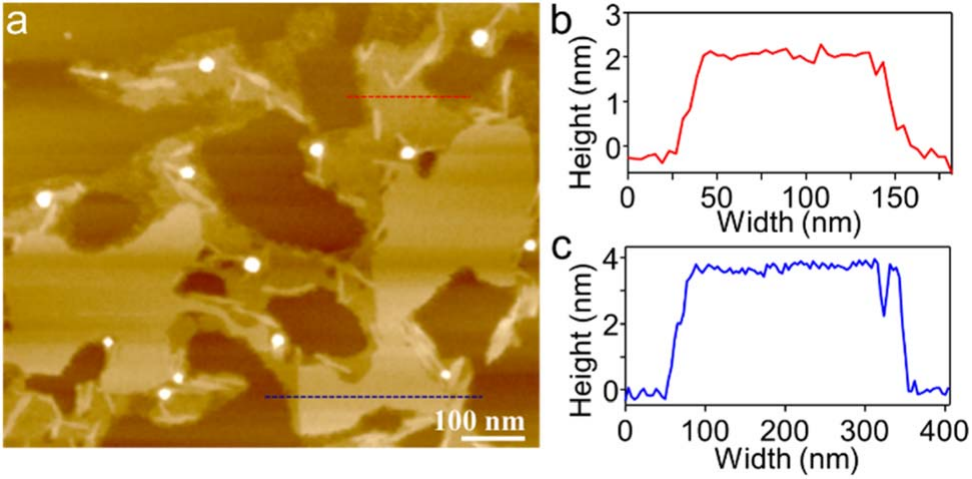
(a) AFM image of A_4_-NS on HOPG and (b and c) the corresponding height profile as indicated in red and blue lines in (a).

To visualize the formation of 2D radial-π-stacks, we investigated the morphology of A_4_ aggregates by atomic force microscopy (AFM). The presence of terraces and step-edges of overlapping nanosheets with lateral sizes of hundreds of nanometers evidenced the formation of 2D layered structures on highly oriented pyrolytic graphite (HOPG) surfaces (Fig. 3 and S2†). The height profile demonstrated two types of domains differing in heights (1.9 and 3.8 nm). The thickness of the short domains is well correlated with the height of the nanopillar molecules (Scheme 1a). This correlation indicates that the alkyl chains at the imide positions interact with the HOPG substrate, while the A_4_ nanosheets (A_4_-NS) adopt an upright orientation on the surface, rather than lying flat. The height of the tall domains is twice that of the short domains, which can be attributed to a bilayer arrangement. Such a tendency to form bilayer structures is not surprising.^49^

### Pre-latticed 2D cavities serving as templates to orchestrate ordered arrangements of absorbed guest molecules

In order to complement the conjugated 2D carbon network structure, recent advancements have involved the preparation of monolayer polymeric C_60_, where the molecules are covalently bonded to each other within a plane.^50–52^ The polymeric C_60_ forms closely packed hexagonal and tetragonal arrays, thereby exhibiting distinct properties associated with these particular arrangements. Conversely, when C_60_ is absorbed onto surfaces, the formation of closely packed molecular monolayers is suppressed due to the weak C_60_–C_60_ interaction and C_60_–substrate interaction.^53^ To prevent lateral diffusion of arranged C_60_ molecules on the surface, a highly effective approach involves using flatter macrocycles to create a 2D periodic array of molecular pits, which serves as the basis for selectively arranging C_60_ on the surface.^54–56^ In these precisely arranged 2D arrays, with the aid of macrocycles as an additional molecular layer, each C_60_ molecule is isolated independently, without any interactions between C_60_ molecules or between C_60_ and the surface. This arrangement of C_60_ molecules is not ideal for electronic applications that require the movement of electrons between them in a bulk material, which is crucial for the performance of devices such as organic field-effect transistors.^57^

**Fig. 4:**
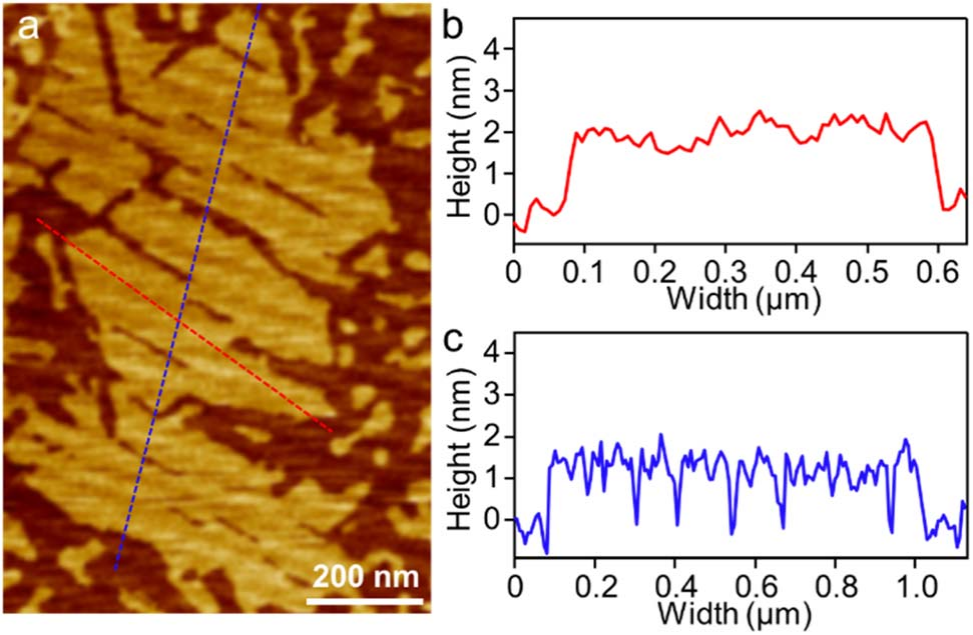
(a) AFM image of (A_4_-C_60_)-NS on HOPG and (b and c) the corresponding height profile as indicated in red and blue lines in (a).

**Fig. 5:**
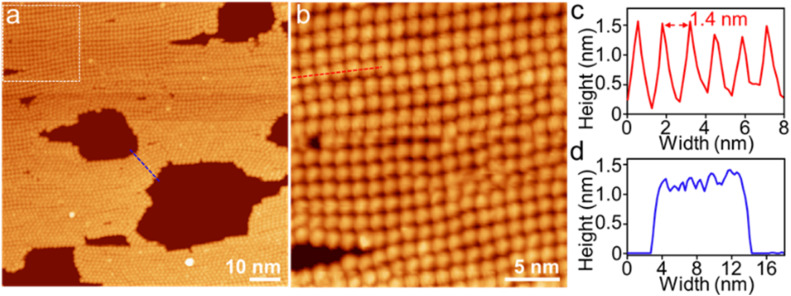
A typical large-scale UHV-LT-STM image (a) of an (A_4_-C_60_)-NS array on a HOPG surface; (b) the images of the same regions after expansion; (c) the distance profile taken from a red line in (b); (d) the height profile taken from a blue line in a).

[4]C-NDTIs have been employed as a molecular encapsulation for C_60_ one-on-one through strong concave–convex π–π interaction.^42^ The 2D arrays of [4]C-NDTIs may also make the incoming C_60_ arrange in a regular pattern. Accordingly, a Tol/*n*-BuOH solution of A_4_ (*c* = 10^−4^ M) and a toluene solution of C_60_ (*c* = 10^−3^ M) were mixed in equimolar proportions and deposited onto HOPG and examined using AFM (Fig. 4 and S4†). Sheets with uniform height relative to the HOPG substrate (∼1.8 nm) and lateral dimensions ranging from two hundred nanometers to five hundred nanometers were observed. Based on the aforementioned findings, these objects were identified as single-layer sheets. Owing to the remarkable electronic conductivity of C_60_, it can serve as a molecular wire bridging two electrodes in break junction studies. As A_4_-NS adopt an upright orientation on the surface, the C_60_ in the A_4_ cavity come into efficient contact with the surface. Consequently, by using A_4_-NS as a template, a periodic monolayer array of C_60_s could be attained and imaged, which can in return reveal the arrangement of A_4_ in the nanosheets. A well-developed 2D regular pattern of a square lattice over a large surface area of 90 × 90 nm^2^ was observed by scanning tunneling microscopy (STM) (Fig. 5 and S5†). This arrangement contrasts with the typical hexagonal close packing of pure C_60_ at room temperature and ultimately leads to convex–convex π–π interactions between A_4_-C_60_ propagated in a 2D plane throughout the crystal lattice. The bright features in Fig. 5a and b correspond to individual C_60_ molecules sited within A_4_. The horizontal distance between neighbouring C_60_ molecules is about 1.4 nm (Fig. 5c and d), which is in good agreement with the diameter of A_4_ and the SAXS result (Fig. 1b and 2c). The thickness of (A_4_-C_60_)-NS is approximately 1.3 nm (Fig. 5d), which is much larger than the diameter of C_60_ (∼0.75 nm).^56,58^ With the aid of 2D π-stacks, we have achieved the successful fabrication of stable monolayer 2D [4]C-NDTI-C_60_ arrays as a consequence of multiple π–π interactions. The presence of these stable, large-size 2D C_60_ arrays opens up exciting opportunities for further comprehensive characterization and exploration of their potential applications as integrated organic devices.

### Excited-state dynamics of A_4_ and A_4_-NS

**Fig. 6:**
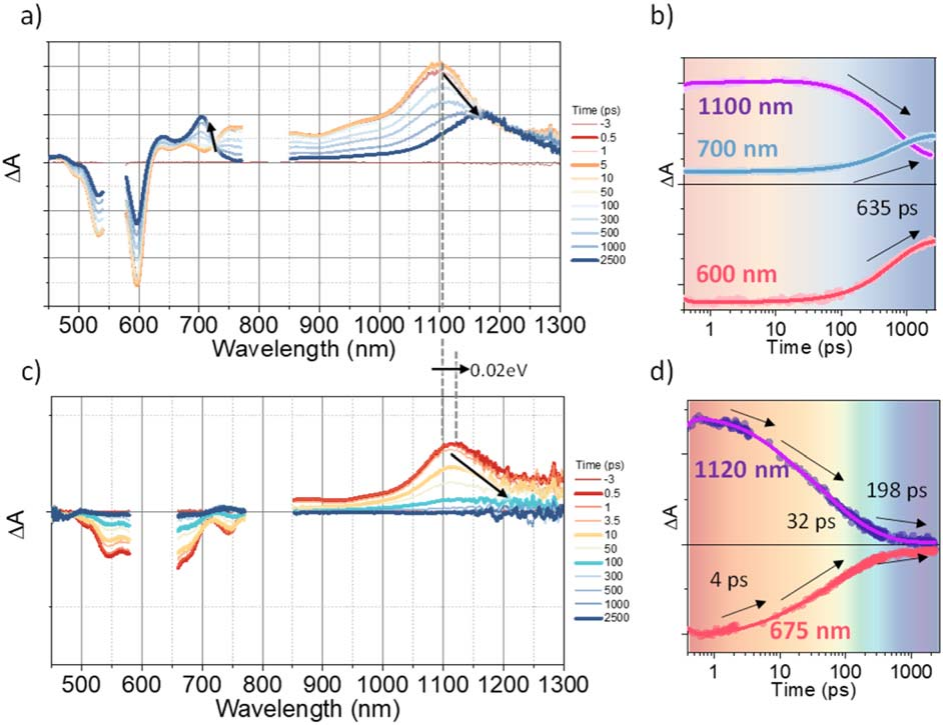
(a and b) The TA results of A_4_ in Tol: a) the representative TA spectra and b) TA kinetics (the sample was pumped with 500 μW (50 nJ per pulse) at 550 nm); (c and d) the TA results of A_4_-NS in Tol/*n*-BuOH solution (1/49, v/v, 10 μM). (c) the representative TA spectra and (d) TA kinetics (the sample was pumped with 100 μW (10 nJ per pulse) at 630 nm). (The pump scattering is blocked for clarity. The arrows are used to guide the spectral/temporal evolution for eyes.).

**Fig. 7:**
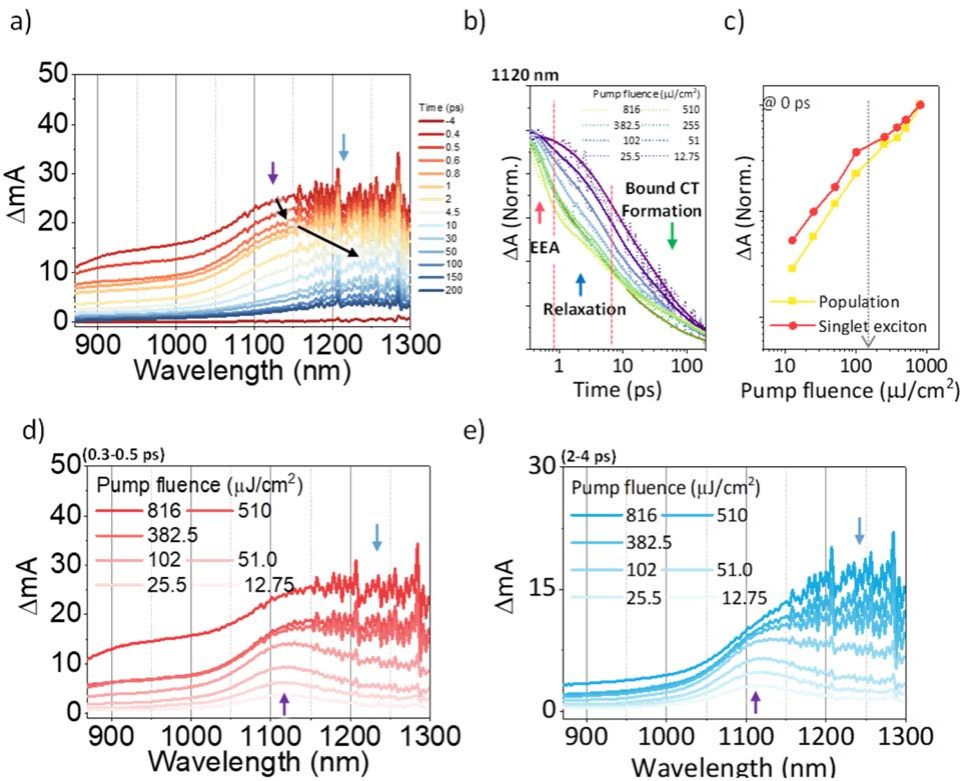
(a) The representative TA spectra of A_4_-NS in Tol/*n*-BuOH solution (1/49, v/v, 10 μM, and the sample was pumped with 3200 μW (320 nJ per pulse) at 630 nm); (b) the normalized TA kinetics at ESA for S_1_ (1120 nm); (c) the normalized TA signal plot as a function of pump fluence at near 0 ps the arrow indicates the threshold of photon density (1.09 × 10^18^ cm^−3^); (d and e) based on the TA kinetics analysis, the temporal regions were categorized: (region 1) prompt (0.3–0.5 ps, (d)) and (region 2) after the annihilation process (2–4 ps, (e)) (the other regions are shown in Fig. S8.†).

To investigate the exciton dynamics of 2D π-stacks, we have carried out femtosecond transient absorption (TA) measurements (Fig. 6, S6 and S7†). First, we investigated A_4_ in its monomeric form. In Tol, the TA spectra indicate the clear red-shift of excited-state absorption (ESA) peaks (1100 to 1150 nm) with a time constant of 635 ps (red to blue line in Fig. 6a), suggesting the evolution of the initial S_1_ state into a charge transfer (CT) state. The formation rate constant (*k*_CT_) is strongly correlated with the solvent polarity (*k*_CT_ = (490 ps)^−1^ in chloroform (CF), (459 ps)^−1^ in dichloromethane (DCM), and (369 ps)^−1^ in benzonitrile (BCN)), supporting that the newly emerging peak at 1150 nm could be assigned to the CT state (Fig. S6†). Such a CT character could have originated from the strong interaction between fused thiophene and NDI units.^59,60^ On the other hand, the excited state dynamics of A_4_-NS shows a sharp contrast with A_4_. Here, we note that a pump fluence of 12.0 μW cm^−2^ was used to exclude the exciton–exciton annihilation (EEA) process. The initial TA spectrum shows that the ESA peak becomes red-shifted by ∼0.02 eV compared to that of A_4_ (1100 to 1120 nm), which can be attributed to the delocalized S_1_ state of A_4_-NS (red line in Fig. 6c). After the minor structural relaxation process (4 ps), the bound charge transfer (BCT) state showing a distinct EAS band at 1200–1300 nm is evolved with a time constant of 32 ps, followed by triplet formation with a time constant of 198 ps (Fig. 6). Interestingly, the novel 2D-nanosheet framework shows characteristic features at high pump fluence (>100 μW cm^−2^) (Fig. 7). The pump-fluence dependent TA results not only show a clear exciton–exciton annihilation (EEA) process but also a striking difference of initial TA spectra between low and high fluences (Table S1† and Fig. 7b and d). The EEA process was observed with a time constant of 400 fs. Considering that EEA is a second order process, *i.e.*, diffusion-limited process, such an ultrafast annihilation process infers the short diffusion length of initial exciton (see ESI Note 1†). Moreover, the initial TA spectra at high pump fluence show a prominent band at 1200–1300 nm, suggesting that the prompt annihilation process generates the BCT state (Fig. 7d). After the annihilation process is over, TA spectra clearly show the prominent BCT contribution in the ESA band at high pump fluences (Fig. 7e). This implies that the singlet exciton annihilation process induces an additional channel for BCT formation, which is reminiscent of multiple relaxation pathways of P3HT polymer towards charge generation.^61,62^ After the EEA process is over, the BCT state is completely formed with 4 and 30 ps (Fig. S8†), which further evolves into a triplet state with ∼200 ps (Fig. 9a). Nevertheless, the absence of pump-fluence dependence at 1250 nm suggests that the BCT state is trapped rather than transported toward the stabilized site (Fig. S9†), which is presumably attributed to the rigid geometry of A_4_-NS. Finally, followed by analysis of Chen *et al.*,^61,62^ we show the relative intensity plot of the TA signal at 0 ps along with the pump flux, which can approximately present the averaged interaction radius of the initial excitons (*i.e.*, the size of delocalized exciton, see ESI Note 2†). An averaged interacting radius is estimated to be ∼6 to 7 nm from the threshold of photon density, suggesting that 5–6 units of A_4_ in A_4_-NS are initially delocalized.

### Energy-funneling and CT formation

**Fig. 8:**
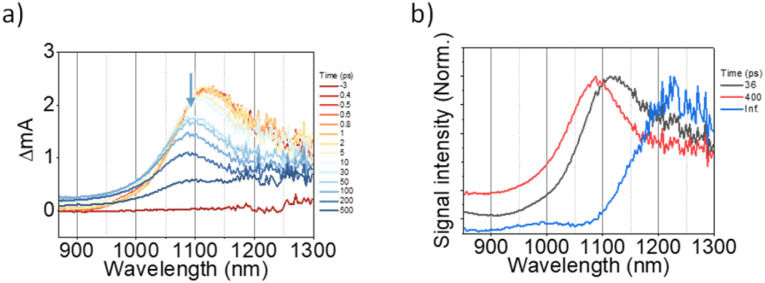
(a) The representative TA spectra of (A4 : A4-PCBM)-NS in Tol/*n*-BuOH solution (Tol/*n*-BuOH = 1/49, v/v, 10 μM, at 12.75 μJ cm^2^); (b) the normalized evolution-associated spectra.

The 2D network of light-harvesting complex II (LHCII) and reaction center-light-harvesting complex I (RC-LHCI) forms an efficient funnel that directs the excitation energy from LHCII to RC-LHCI. This trait inspired us to introduce the A4-PCBM (phenyl-C_61_-butyric acid methyl ester)^63^ inclusion complex as an analogue to the RC-LHCI core in A_4_-NS to investigate the photophysical behaviors of the 2D aggregate. Accordingly, a 9 : 1 mixture of (A4 : A4-PCBM)-NS (molar ratio) was examined to estimate whether the diffusion of free charges is feasible within the 2D architecture. We note that the A_4_-PCBM complex shows the formation of anion species of PCBM at 1060 nm^64,65^ with a time constant of 5.0 ps (Fig. S12†). Interestingly, (A4 : A4-PCBM)-NS shows a striking difference from the self-trapping of A_4_-NS (Fig. 8).

**Fig. 9:**
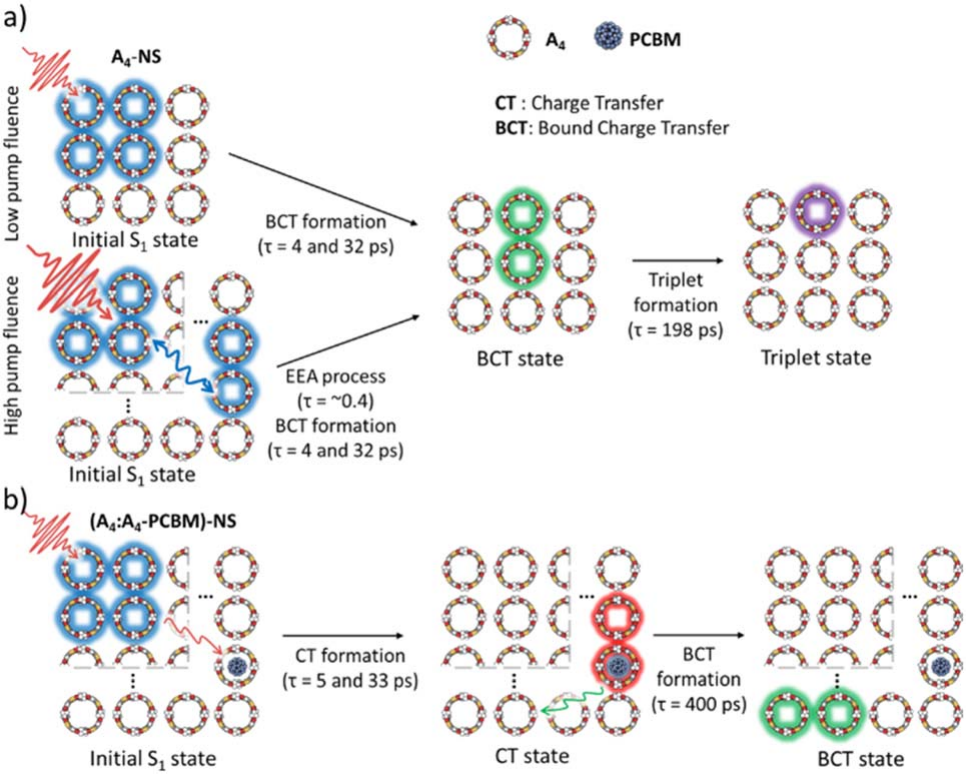
Schematic diagram summarizing the excited-state dynamics of (a) A_4_-NS and (b) (A4 : A4-PCBM)-NS.

Given that the initial ESA band at 1120 nm is the same as that of A_4_-NS (black line in Fig. 8b), we suggest that the A_4_-NS region is initially excited. Nevertheless, the ESA band of A_4_-NS becomes blue-shifted toward that corresponding to the radical anion of PCBM (1060 nm) with the time constants of 5.0 and 33.3 ps, suggesting free CT exciton (CTE) formation (red line in Fig. 8b). This result suggests that the A_4_-NS domain absorbs the light and subsequently the exciton moves towards A_4_-PCBM binding sites to generate CTE along with the energy gradient which comes from PCBM encapsulation (Fig. 9b). The CTE slowly proceeds to the BCT state with 400 ps (Fig. 8b and S13†), which is comparable to other systems (Table S2†). Considering that the BCT state is formed with 30 ps in A_4_-NS, such 10 times slowdown hints at the diffusion of free CT excitons toward the trap site consisting of A_4_-NS.^62^ Due to the abilities of concentrating excitation energy by energy pooling from empty cavities to occupied sites, the 2D radial-π-stacks can be used for guest inclusion for sensing or as nanoreactors.^66^

## Conclusions

In conclusion, we have presented a novel strategy for constructing novel 2D radial-π-stacks based on [4]C-NDTIs through “exo-wall” convex–convex π–π interactions in solution. These special 2D π-stacking structures exhibit a high degree of order and long-range periodicity in solution and display both intra- and inter-ring couplings in a 2D plane. As a 2D template, these 2D π-stacks possess pre-latticed cavities capable of accommodating guest molecules. Through our spectroscopic investigations, we have revealed a photophysical mechanism where light absorption by the 2D π-stacks initiates the delocalization of excitons within the 2D plane, efficiently channeling the energy towards the core complexes of [4]C-NDTI-PCBM. These characteristics are reminiscent of the elegant light harvesting systems in purple bacteria. In light of recent advances in the synthesis of nanoring- and nanobelt-shaped π-systems, our findings are expected to inspire innovative approaches in exploring novel 2D π-stacking materials possessing unique topologies and unparalleled functional properties.

## Data availability

All the data supporting this article have been included in the main text and the ESI.†

## Author contributions

F. S. and Y. H. carried out the experiments, analyzed the data, and wrote the manuscript. G. Z., K. W. and Z. C. carried out the experiments. J. K. and H. Z. assisted in the analysis. D. K. and J. L. designed the project, analyzed the data, and revised the manuscript.

## Conflicts of interest

There are no conflicts to declare.

## Supplementary Material

SC-015-D4SC00195H-s001
