# Supplementary material for: Two-dimensional radial-π-stacks in solution
Source: Chem Sci. 2024 Mar 11;15(15):5604–11. doi: 10.1039/d4sc00195h (PMC11023034; doi:10.1039/d4sc00195h)
Supplement: SC-015-D4SC00195H-s001 [file SC-015-D4SC00195H-s001.pdf]

Electronic Supplementary Material (ESI) for Chemical Science.
This journal is © The Royal Society of Chemistry 2024

Two-Dimensional Radial-π-Stacks in Solution

Feng Su,[a,†] Yongseok Hong,[b,†] Guilan Zhang,[a] Kongchuan Wu,[a] Juno Kim,[b] Zhi Chen,[c] Hui-Jun Zhang,[a] Dongho Kim[b,d*] and Jianbin Lin[a*]

[1]Department of Chemistry, College of Chemistry and Chemical Engineering, MOE Key Laboratory of Spectrochemical Analysis and Instrumentation, Xiamen University, Xiamen 361005, P. R. China.

[2]Department of Chemistry, Yonsei University, Seoul 03722, Korea.

[3]College of Chemistry and Environmental Engineering, Shenzhen University, Shenzhen, 518060, China.

[4]Division of Energy Materials, Pohang University of Science and Technology (POSTECH), Pohang 37673, Korea.

*Corresponding Authors: dongho@yonsei.ac.kr; jb.lin@xmu.edu.cn.

[†]F. Su and Y. Hong contributed equally to this work.

1. General information

General Methods.

Unless otherwise noted, all reagents were obtained from commercial suppliers and used without further purification. Absorption spectra and fluorescence spectra were recorded on a Thermo Scientific Evolution 300 UV/Vis spectrophotometer and Hitachi F-4500, respectively. Circular dichroism (CD) spectra were obtained on a spectropolarimeter (JASCO, Jasco-1500).

The dynamic light scattering (DLS) was measured at an angle of 45 on a standard laser light scattering spectrometer (BI-200SM) equipped with a BI-9000 AT digital time correlator (Brookhaven Instruments, Inc.). A Mini-L30 diode laser 30 mW power laser was used, operating at a wavelength of 637 nm. For evaluation of the data, the DTS (Nano) program was used. The mean positions of the peaks in the number-hydrodynamic diameter (d_H) distribution were taken for data representation. The hydrodynamic diameter (d_H) of the particles is calculated from the diffusion coefficient (D) using the Stokes-Einstein equation $D = k_B T/3\pi \eta d_H$, where T is absolute temperature, η is viscosity of the solvent and k_B is the Boltzamann constant.

Self-assembly Experiments.

A_4 or **B_4** in toluene (60 μL, 5 × 10^{-4} M) was injected into *n*-butanol (1/49, *v*/*v*, 2940 μL) to achieve a final concentration of 10 μM. Subsequent measurements were conducted in time.

Atomic Force Microscopy (AFM).

AFM imaging was carried out on a Bruker Dimension Icon Atomic Force Microscope. The sample was dissolved in toluene/*n*-BuOH (2/98) and diluted to 10^{-4} M. The resulting solution was introduced onto fresh HOPG surface by drop casting, and stood at room temperature for another 1 minute. Then the surface was dried with Kimwipes, and dried with N_2 gas. Silicon cantilever tip with spring constant of around 0.1 N/m was used for the experiments.

Ultra-High-Vacuum Low-Temperature Scanning Tunneling Microscope (UHV-LT-STM).

Sample was dissolved in toluene/*n*-BuOH (2/98) at a concentration of 10^{-4} M, and 1 equivalent C_{60} in toluene was added. Onto the freshly HOPG surface, 0.2 mL of the solution was deposited by drop casting, and stood for 10 seconds. Then the surface was dried slightly with Kimwipes. After the samples were dried at room temperature with N_2 gas, STM imaging was conducted on a LT-STM-AFM-BO scanning tunneling microscope (CreaTec Fischer & Co. GmbH) at 77 K. An electrochemically etched polycrystalline tungsten wire was used for the STM tip. The obtained STM images were analyzed by WSxM5.0 software.

Transient Absorption Spectroscopy.

A Ti:sapphire laser system (Spectra-Physics, Spitfire) provides 30 fs, 350 μJ pulses at 800 nm with 10 kHz repetition rate. The output beam is divided by a beam splitter with an equivalent ratio. A pulse of 175 μJ is used to pump an optical parametric amplifier (TOPAS, Light Conversion). The generated OPA pulses had a pulse width of ~ 50 fs and an average power of 100 mW in the range 280-2700 nm which were used as pump pulses. White light continuum (WLC) probe pulses were generated using a sapphire window (3 mm of thickness) by focusing of small portion of the fundamental 800 nm pulses which was picked off by a quartz plate before entering to the OPA. After the measurements, we carefully checked absorption spectra of all compounds to detect if there were artifacts due to degradation and photo-oxidation of samples. HPLC grade solvents were used in all measurements. The three-dimensional data sets of ΔA versus time and wavelength were subjected to singular value decomposition and global fitting to obtain the kinetic time constants and their associated spectra using Surface Xplorer software (Ultrafast Systems)

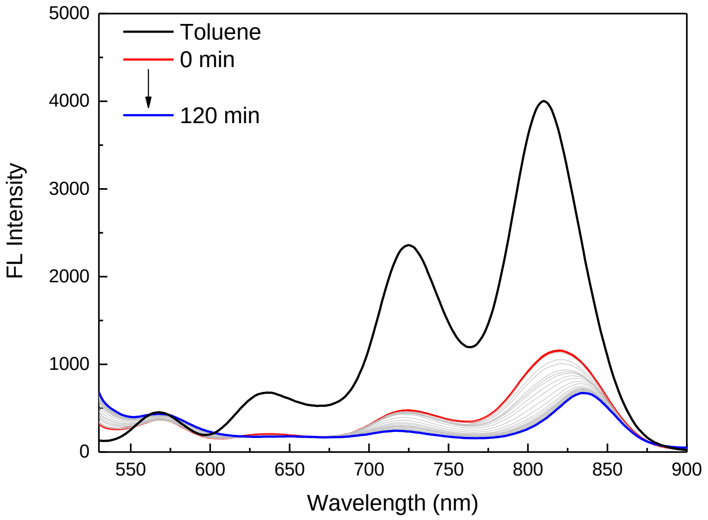

Figure S1. Time-dependent fluorescence spectra. (Tol/*n*-BuOH = 1/49, *v*/*v*, 10 μM)

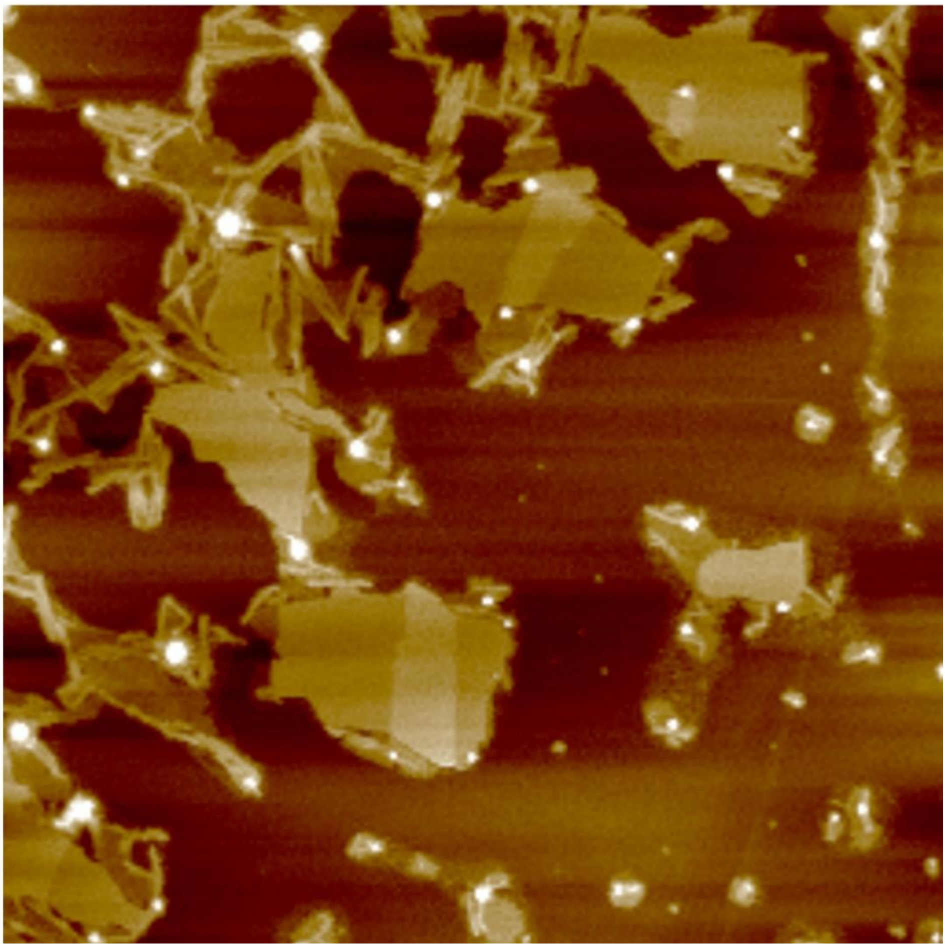

Figure S2. AFM image of the **A$_4$-NS** on HOPG.

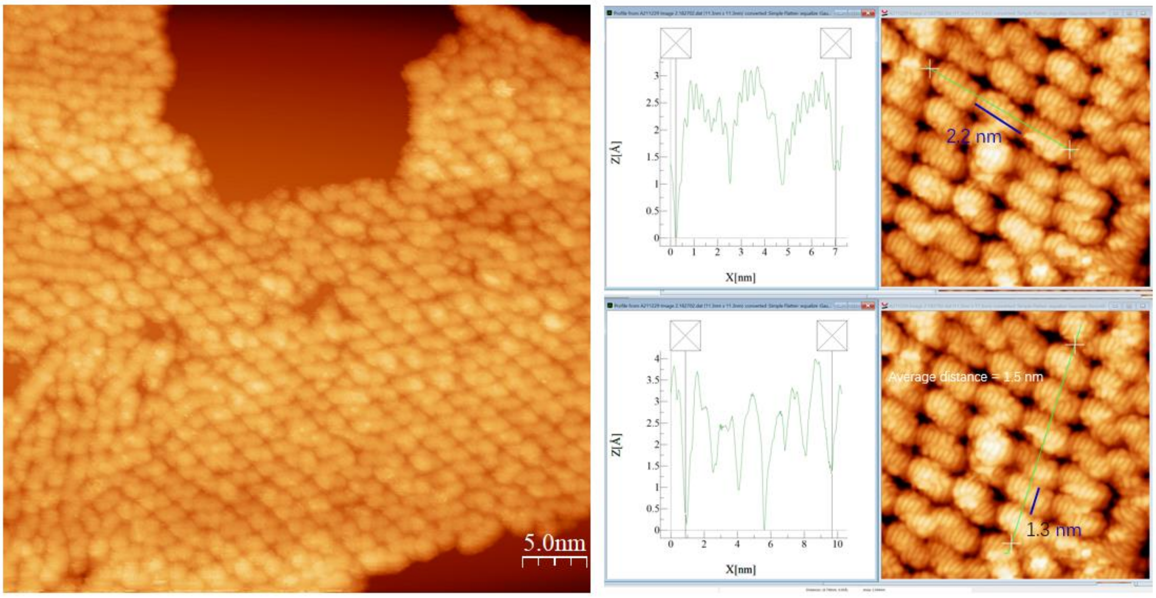

Figure S3. UHV-LT-STM image of **A₄** in MCH on a HOPG surface.

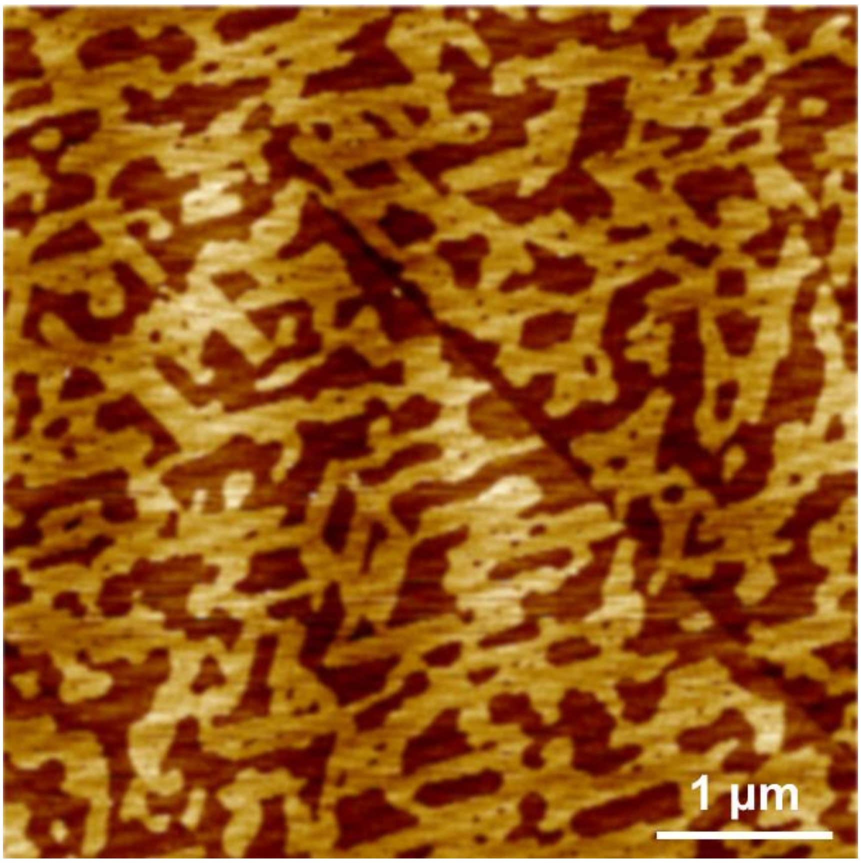

Figure S4. AFM image of the **(A₄-**C_{60}**)-NS** on a HOPG surface.

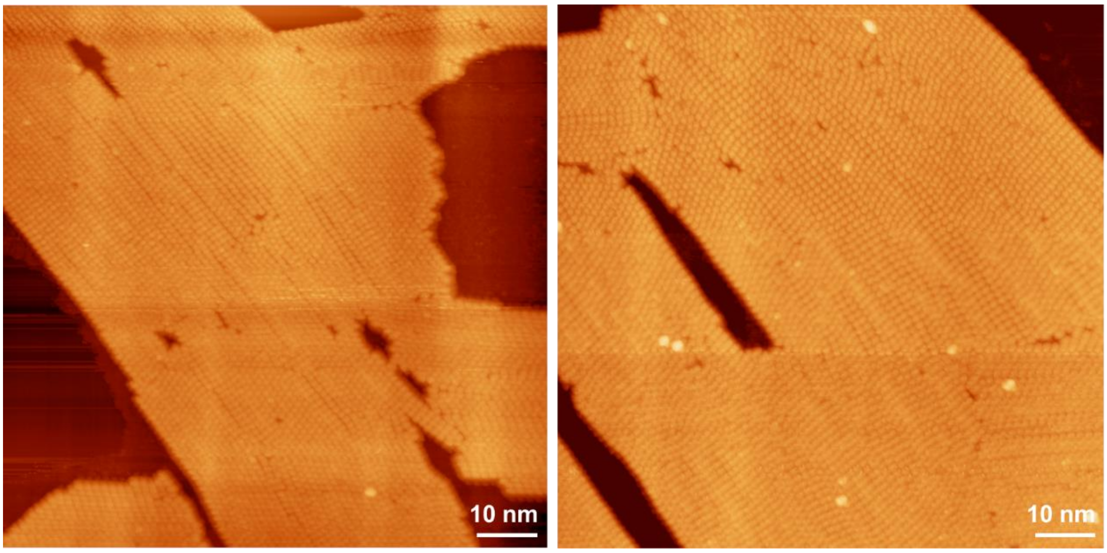

Figure S5. UHV-LT-STM image of an **(A_4-C_{60})-NS** array on a HOPG surface.

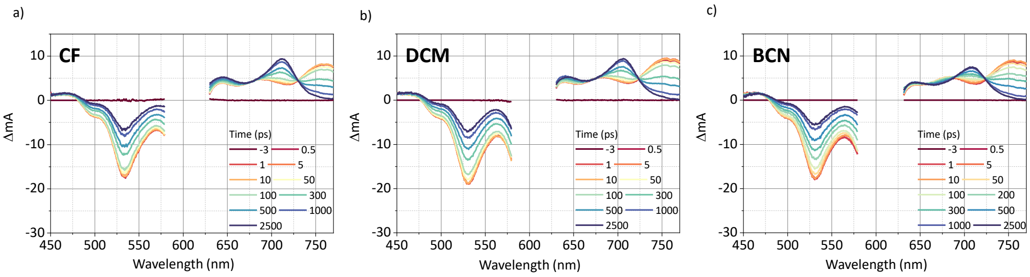

Figure S6. Representative TA spectra of **A_4** in chloroform (a), dichloromethane (b), and benzonitrile (c).

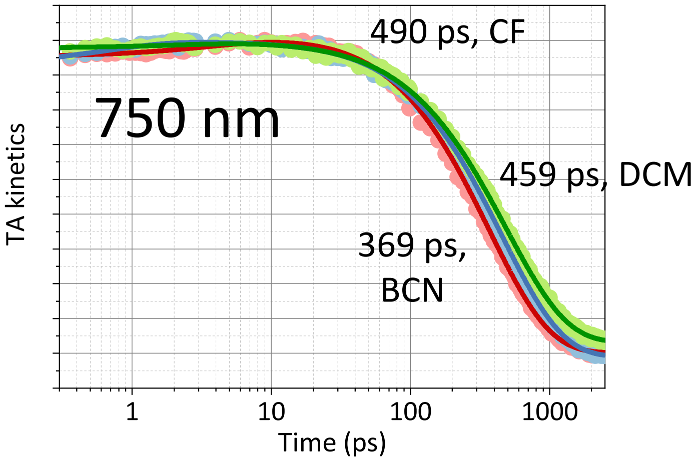

Figure S7. Normalized TA kinetics of **A_4** at 750 nm in chloroform (green), dichloromethane (blue), and benzonitrile (red).

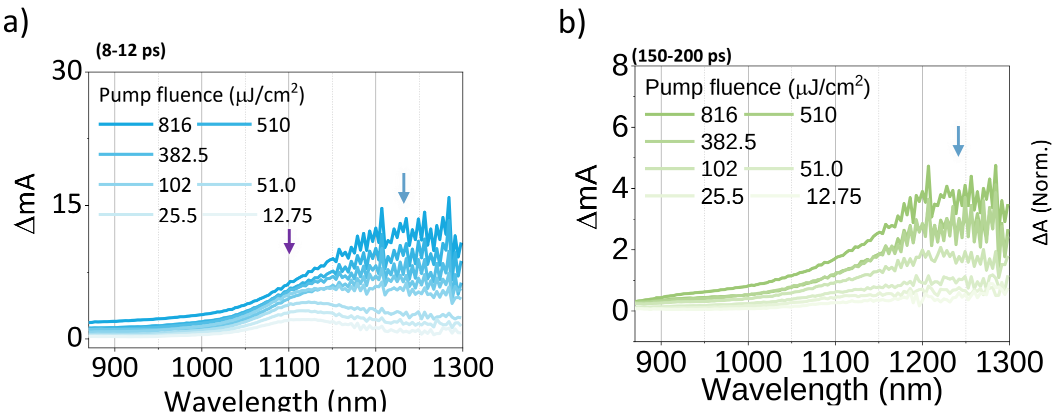

Figure S8. a-b) Based on the TA kinetics analysis, the temporal regions were categorized: (region 3) after relaxation (8-12 ps, a), (region 4) after BCT formation process (150-200 ps, b). (Tol/*n*-BuOH = 1/49, *v*/*v*, 10 μM)

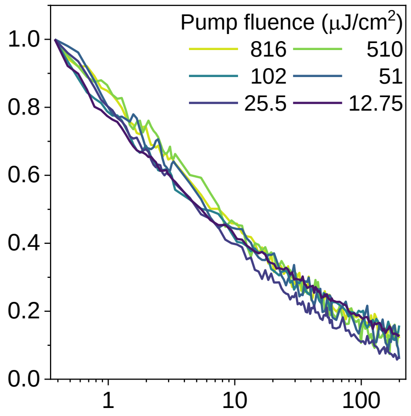

Figure S9. The normalized TA kinetics at ESA for BCT. Here, we show the absence of pump fluence dependence of excited state species covering 1200-1300 nm, suggesting the excited-state species is immobile. Thus, this state can be assigned to BCT rather than FCT. (Tol/*n*-BuOH = 1/49, *v*/*v*, 10 μM)

Supplementary note 1

The effect on singlet-singlet annihilation process

Table S1 Fitting parameters of pump-fluence dependent TA kinetics (probed at 1100 nm).[a]

Pump fluence ($\mu W/cm^2$)	0.4 ps[a]	4 ps	30 ps	200 ps
12.75	0	37.1%	38.8%	24.1%
25.5	24.2%	35.0%	23.6%	17.2%
51	35.4%	31.9%	21.2%	11.5%

102	41.1%	31.5%	13.2%	14.1%
255	52%	26.5%	14.0%	7.7%
382.5	51.3%	26.3%	10.1%	12.3%
510	54.8%	25.1%	7.4%	12.6%
816	63.6%	19.9%	4.8%	11.7%

[a] The TA kinetics were fitted by multiexponential function convolved with IRF (0.25 ps). [b] assigned the annihilation process.

Taking pump-fluence dependent TA results, we propose that the initial exciton diffusion enables the multiplication of bound CT exciton in that EEA process is second-order reaction, which is further investigated by detailed analysis of TA spectra and modeling. Considering that the annihilation process is second-order process and diffusion-limited process, the analysis of EEA kinetics provides an additional information on how long the exciton can diffuse. We assume that initial exciton diffuses along with an axis (1D model) because of the strong J-type dipole-dipole interaction. The diffusion rate constant can be estimated from the following equations.[1]

Table S2 Excited-state time-scale in recently reported SSA systems.

(ps)	τ_1	τ_2	τ_3	τ_4	τ_5
2D COF [a]	**4 ps**; Excimer formation (EEA)	160 ps; Excimer relaxation	> 3ns; Excimer decay		
Porphyrin nanoring [b]	**0.3 and 2.6 ps**; intramolecular vibrational energy redistribution, vibrational cooling, electronic energy transfer (EEA)		35 ps; exciton motion	250 ps; singlet-to-triplet relaxation	> 3 ns; Exciton lifetime
PDI H-aggregate [c]	< 50 fs; Exciton formation	0.8 ps EEA	40-50 ps; excited multimer formation	~300 ps; structural rearrangement	> 40 ns; exciton lifetime

[a] *J. Am. Chem. Soc.*, 2020, **142**, 14957–14965.
[b] *J. Phys. Chem. C,* 2020, **124**, 18416–18425.
[c] *Chem. Sci*., 2013, **4**, 388-397.

Supplementary note 2

While TA spectra at 0 ps can be underestimated due to the limitation of instrumental resolution function (~0.25 ps), these results can minimize the distortion of TA signal by photo-induced processes such as singlet exciton annihilation process or singlet exciton Forster energy transport. Thus, we assume that the initial exciton size contributes to the pump-flux dependence of TA signal at 0 ps (Fig. 6e).

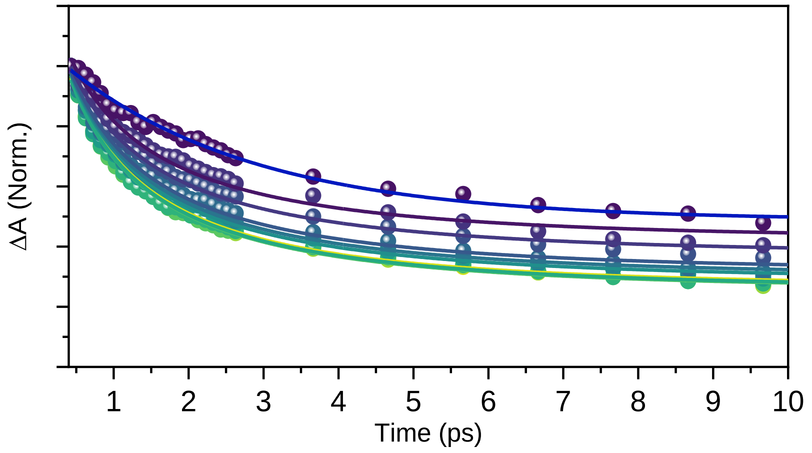

Figure S10. (a) TA kinetics at 1100 nm fitted by equation S1.

$$S_1 = \frac{A_0 e^{-t/\tau_1}}{1+A_0\gamma(1-e^{-t/\tau_1})} \cdots\cdots \text{(S1)}$$

, where r is second order decay rate constant, τ_1 is singlet exciton lifetime, A0 is initial S1 population. According to previous works, we derivative the diffusion constant (D) and diffusion length (L) by using r.

$$\gamma = 4\pi(2D)R \cdots\cdots \text{(S2)}$$

$$L = \sqrt{D\tau} \cdots\cdots \text{(S3)}$$

Here, we assume that the initial exciton lifetime is 16 ps, which is weight averaged lifetime obtained from TA kinetics at the lowest pump fluence. Moreover, we fitted the TA kinetics up to 10 ps due to the following experimental limitations: (1) The distortion of TA kinetics at high pump fluence by bound CT exciton population that is produced by EEA or prompt annihilation and (2) large spectral overlap of S1 and CT bands, which complicates analysis of S1 population. Therefore, pump-fluence dependent TA kinetics at 550 and 1150 nm are plotted by using equation 1 in the range of 0-10 ps to minimize the distortion of S1 population. The r value is estimated to $9.3*10^{-10}$ cm^3/s. By assuming that the annihilation radius (R) of 2D aggregate is similar to other organic materials (~1 nm), the diffusion constant can be estimated to 0.0017 cm^2/s, corresponding to diffusion length of 0.26 nm. Compared to other organic materials, where the diffusion constants lie between 0.1 to 0.001, the diffusion constant is small, which is attributed to the large density of bound CT state acting as trap site.

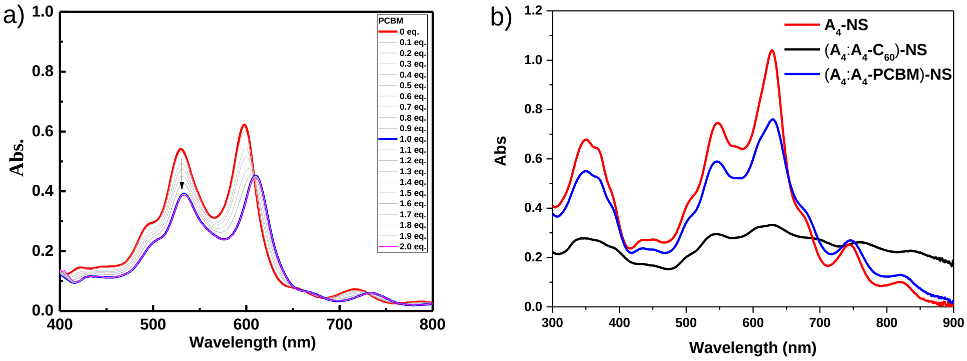

Figure S11. a) UV-Vis titration experiment for **A4** ⊃ **PCBM** in toluene (4 μM in toluene); b) UV-Vis-NIR absorption spectra of three nanosheets obtained after 2 hours (Tol/*n*-BuOH = 1/49, *v*/*v*, 10 μM).

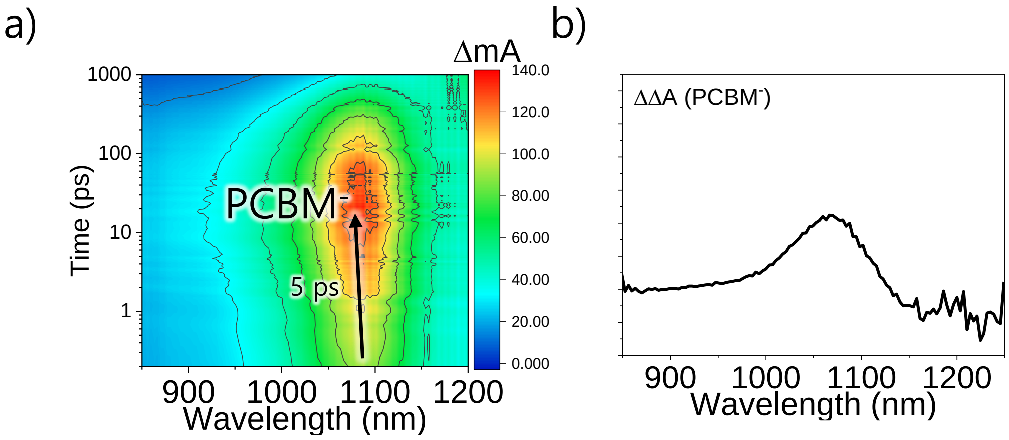

Figure S12. a) TA contour map of **A4-PCBM** in Tol; b) The TA difference spectrum ΔΔA (blue) between the initial (500 fs) and CT (20 ps) states.

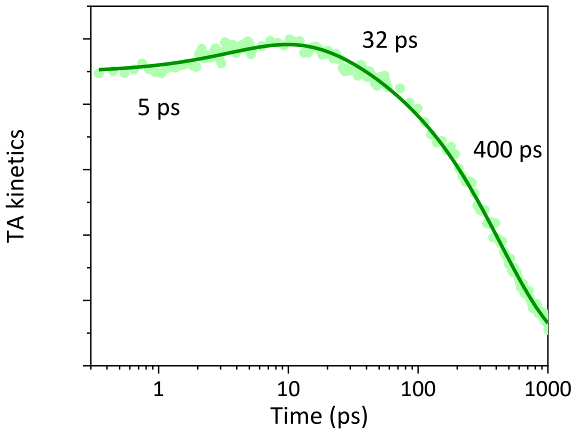

Figure S13. Normalized TA kinetics of (**A4**:**A4-PCBM)-NS** at 1080 nm.

Reference:

1. Flanders, N. C.; Kirschner, M. S.; Kim, P.; Fauvell, T. J.; Evans, A. M.; Helweh, W.; Spencer, A. P.; Schaller, R. D.; Dichtel, W. R.; Chen, L. X., Large Exciton Diffusion

Coefficients in Two-Dimensional Covalent Organic Frameworks with Different Domain Sizes Revealed by Ultrafast Exciton Dynamics. *J. Am. Chem. Soc.* **2020**, *142* (35), 14957-14965.
